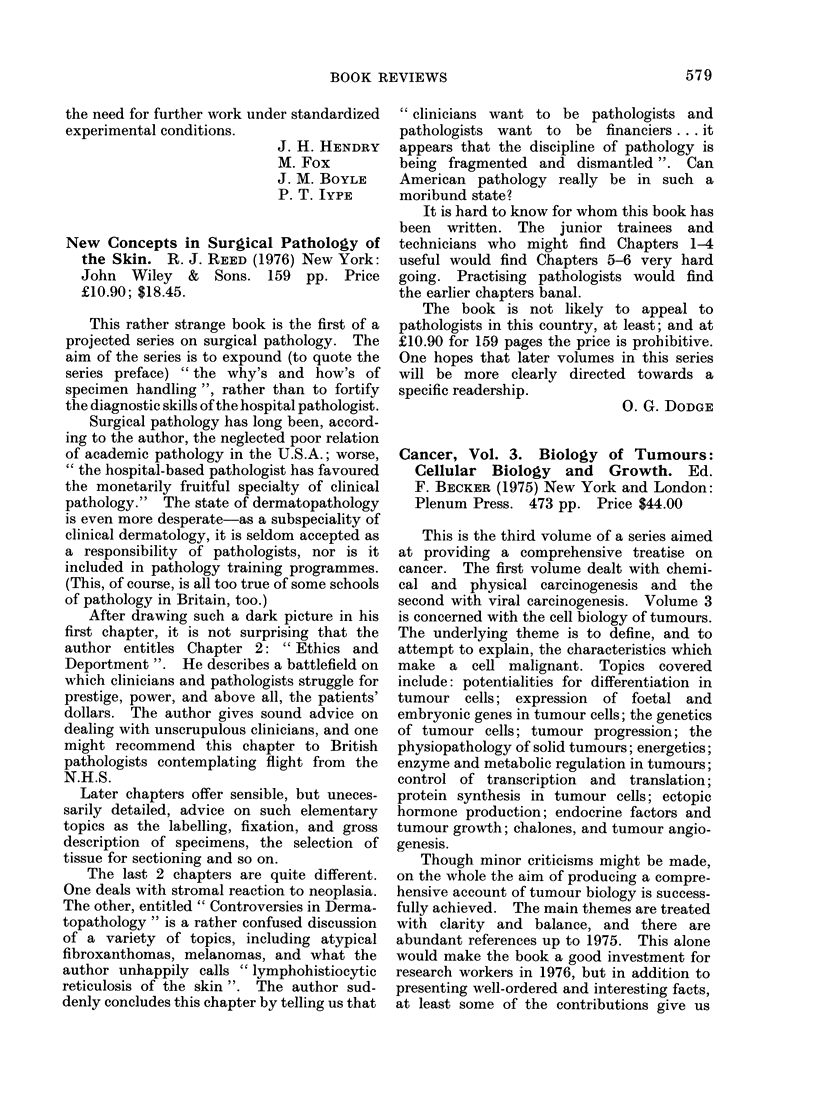# New Concepts in Surgical Pathology of the Skin

**Published:** 1976-11

**Authors:** O. G. Dodge


**New Concepts in Surgical Pathology of the Skin.** R. J. Reed (1976) New York: John Wiley & Sons. 159 pp. Price £10.90; $18.45.

This rather strange book is the first of a projected series on surgical pathology. The aim of the series is to expound (to quote the series preface) " the why's and how's of specimen handling ", rather than to fortify the diagnostic skills of the hospital pathologist.

Surgical pathology has long been, according to the author, the neglected poor relation of academic pathology in the U.S.A.; worse, " the hospital-based pathologist has favoured the monetarily fruitful specialty of clinical pathology." The state of dermatopathology is even more desperate—as a subspeciality of clinical dermatology, it is seldom accepted as a responsibility of pathologists, nor is it included in pathology training programmes. (This, of course, is all too true of some schools of pathology in Britain, too.)

After drawing such a dark picture in his first chapter, it is not surprising that the author entitles Chapter 2: " Ethics and Deportment ". He describes a battlefield on which clinicians and pathologists struggle for prestige, power, and above all, the patients' dollars. The author gives sound advice on dealing with unscrupulous clinicians, and one might recommend this chapter to British pathologists contemplating flight from the N.H.S.

Later chapters offer sensible, but unecessarily detailed, advice on such elementary topics as the labelling, fixation, and gross description of specimens, the selection of tissue for sectioning and so on.

The last 2 chapters are quite different. One deals with stromal reaction to neoplasia. The other, entitled " Controversies in Dermatopathology " is a rather confused discussion of a variety of topics, including atypical fibroxanthomas, melanomas, and what the author unhappily calls " lymphohistiocytic reticulosis of the skin ". The author suddenly concludes this chapter by telling us that " clinicians want to be pathologists and pathologists want to be financiers . . . it appears that the discipline of pathology is being fragmented and dismantled ". Can American pathology really be in such a moribund state?

It is hard to know for whom this book has been written. The junior trainees and technicians who might find Chapters 1–4 useful would find Chapters 5–6 very hard going. Practising pathologists would find the earlier chapters banal.

The book is not likely to appeal to pathologists in this country, at least; and at £10.90 for 159 pages the price is prohibitive. One hopes that later volumes in this series will be more clearly directed towards a specific readership.

O. G. Dodge